# Visual Cortex Activity and Retinal Changes in Primary Open-Angle Glaucoma: An fMRI and OCTA Study

**DOI:** 10.3390/medicina62102018

**Published:** 2026-10-19

**Authors:** Austėja Judickaitė, Arminas Zizas, Robertas Petrolis, Algimantas Kriščiukaitis, Saulius Lukoševičius, Alon Harris, Keren Wood Shalem, Ingrida Janulevičienė

**Affiliations:** 1 Department of Ophthalmology, Lithuanian University of Health Sciences, LT-44307 Kaunas, Lithuania; austeja.judickaite@kaunoklinikos.lt (A.J.); arminas.zizas@lsmu.lt (A.Z.); 2 Department of Physics, Mathematics and Biophysics, Lithuanian University of Health Sciences, LT-44307 Kaunas, Lithuania; robertas.petrolis@lsmu.lt (R.P.); algimantas.krisciukaitis@gmail.com (A.K.); 3 Department of Radiology, Lithuanian University of Health Sciences, LT-44307 Kaunas, Lithuania; saulius.lukosevicius@lsmu.lt; 4 Department of Ophthalmology, Icahn School of Medicine at Mount Sinai, New York, NY 10029, USA; alonharris@mssm.edu (A.H.); keren.woodshalem@mssm.edu (K.W.S.)

**Keywords:** glaucoma, visual cortex, functional magnetic resonance imaging, retina, blood vessels

## Abstract

*Background and Objectives*: Glaucoma is a neurodegenerative disease involving retinal ganglion cell loss, optic nerve damage and alterations within the central visual pathway. We evaluated visual cortex activity using functional magnetic resonance imaging (fMRI) in patients with primary open-angle glaucoma (POAG) and its associations with retinal structural and microvascular parameters assessed using optical coherence tomography (OCT) and OCT angiography (OCTA). *Materials and Methods*: This prospective clinical study included 19 patients with POAG and 18 healthy controls. All participants underwent ophthalmological examination, standard automated perimetry (Humphrey 24–2 SITA Standard), swept-source OCT, OCTA of the optic nerve head (ONH) (DRI-OCT Triton, Topcon, Tokyo, Japan), and visual stimulation fMRI (3T Siemens Magnetom Skyra, Siemens Healthineers, Erlangen, Germany). Retinal nerve fiber layer (RNFL) thickness, vessel density (VD), and visual cortex activity were compared between groups. Associations were assessed using Pearson’s or Spearman’s correlation according to data distribution, with Benjamini–Hochberg false discovery rate correction. Unadjusted *p* and adjusted q values are reported. *Results*: RNFL thickness was reduced in POAG patients in all four sectors. VD was lower in every sector of the superficial and deep plexus (all q < 0.05), as was outer retinal signal. Choriocapillaris findings were heterogeneous. RNFL thickness correlated with choriocapillaris VD in two of three corresponding nasal sectors, in POAG patients only. Visual cortex activation was lower in POAG patients (*p* = 0.046; Hedges’ g = −0.68, 95% CI −1.34 to −0.01), but not after adjustment for age (*p* = 0.086). No association between VD and cortical activity survived FDR correction. *Conclusions*: In POAG, choriocapillaris VD tracked RNFL thickness in corresponding nasal sectors, an association not detected in controls. Reduced cortical activation did not persist after adjustment for age, and no retinal–cortical microvascular association was found. These exploratory findings suggest that distinct retinal vascular layers may carry different pathophysiological information and require confirmation in larger cohorts.

## 1. Introduction

Glaucoma, a leading cause of irreversible blindness worldwide, is a chronic progressive neurodegenerative disease characterized by retinal ganglion cell (RGC) loss, optic nerve damage, and irreversible visual field impairment [1]. Increasing evidence supports the concept that glaucoma is not limited to the eye but involves widespread neurodegenerative changes extending throughout the visual pathway and central nervous system (CNS).

Histopathological and neuroimaging studies have demonstrated structural and functional alterations in the lateral geniculate nucleus, visual cortex, and other brain regions associated with visual processing [2]. Functional magnetic resonance imaging (fMRI) has become an important non-invasive tool for investigating cortical activity and functional connectivity in glaucoma patients [3]. Previous studies have reported altered activity within the primary and secondary visual cortices, reduced functional connectivity within visual networks, and abnormalities involving higher-order cortical regions in patients with primary open-angle glaucoma (POAG) [4]. These findings support the hypothesis that glaucoma may represent a broader neurodegenerative disorder involving both ocular and CNS components.

Retinal nerve fiber layer (RNFL) thinning is a hallmark structural feature of glaucomatous damage that correlates with disease severity and visual field loss [1,5]. Optical coherence tomography angiography (OCTA) enables evaluation of retinal and optic nerve head (ONH) vessel density (VD) without contrast agents. OCTA has demonstrated reduced peripapillary and macular VD in glaucoma, suggesting that microvascular impairment accompanies retinal neurodegeneration [6,7]. Because the retina and visual cortex are functionally interconnected structures of the visual pathway, retinal structural and microvascular changes may reflect broader neurodegenerative alterations occurring within the CNS [6,8]. Although both retinal microvascular impairment and cortical functional alterations have been described in glaucoma, they have largely been studied separately, and it remains unknown whether specific retinal vascular layers relate differentially to structural damage and to cortical activity.

The aim of this study was to evaluate visual cortex activity using fMRI in patients with POAG and to investigate its associations with retinal structural and microvascular parameters obtained using OCT and OCTA. Additionally, glaucoma patients were compared with healthy control subjects in order to assess differences in retinal morphology, VD, and cortical functional activity. We prespecified one hypothesis: that RNFL thickness would correlate with VD in anatomically corresponding sectors in patients with POAG. All analyses of VD in relation to cortical activity were exploratory.

## 2. Materials and Methods

This prospective clinical study was conducted in the Ophthalmology and Radiology Departments of the Hospital of Lithuanian University of Health Sciences, Kaunas Clinics. A total of 19 POAG patients and 18 healthy controls were enrolled in this study. The Kaunas Regional Bioethics Committee approved the research protocol (No. BE-2-115). All participants provided written informed consent prior to inclusion in the study.

The sample size was estimated using Altman’s nomogram. Based on previously reported marked differences in visual cortex BOLD responses between patients with primary open-angle glaucoma and healthy controls, a large standardized effect size of 0.95 was assumed. With a two-sided significance level of 0.05 and a statistical power of 80%, the minimum required sample size was estimated to be 18 participants per group. The final study population consisted of 19 patients with POAG and 18 non-glaucomatous healthy controls [4].

The inclusion criteria for the POAG group were a confirmed clinical diagnosis by a board-certified glaucoma specialist, including characteristic changes in the ONH and visual field defects consistent with glaucomatous disease. Healthy control subjects included volunteers with no history of glaucoma or other ocular or systemic diseases that could influence the study outcomes. The exclusion criteria for all participants included pregnancy or breastfeeding, high refractive error (myopia or hyperopia greater than 6.0 diopters, or astigmatism greater than ±3.0 diopters), amblyopia, previous ocular trauma, uncontrolled systemic diseases, a history of allergy to local anesthetics, or any other condition that could potentially bias the study results.

All patients had a clinical diagnosis of POAG with bilateral disease. Visual field loss in the studied eye was graded by the Hodapp–Parrish–Anderson criteria: 10 patients had mild loss, 5 had moderate loss, and 4 had severe loss. All patients had bilateral disease and intraocular pressure was compensated for topical therapy at the time of examination. Median disease duration since diagnosis was 4.0 years (IQR 2.0–8.5; range 0.5–16). All patients were receiving topical intraocular pressure-lowering therapy at the time of examination. Medication regimens were not standardized.

**Figure 1:**
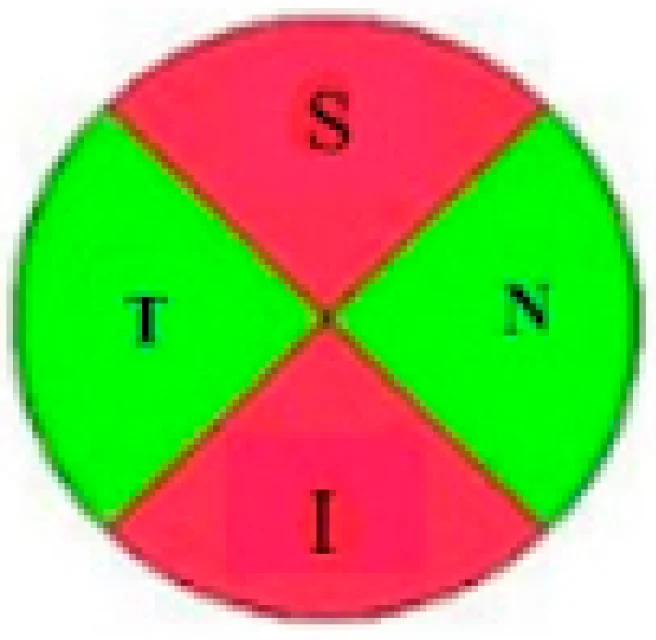
Peripapillary RNFL thickness map (µm) obtained with the OCT 3D Disc scan protocol (6 × 6 mm). The map was divided into four quadrants—superior, inferior, nasal and temporal—for quantitative analysis.

All subjects underwent a standard ophthalmological examination, including best-corrected visual acuity (BCVA) assessment using a Snellen chart, biomicroscopy, ophthalmoscopy, and intraocular pressure (IOP) measurement with Goldmann applanation tonometry. Central corneal thickness (CCT) was measured by pachymetry (Alcon OcuScan RxP, Irvine, CA, USA). Standard automated perimetry 24-2 was performed using the Humphrey Field Analyzer (Carl Zeiss Meditec, 07745 Jena, Germany). The optic nerve and peripapillary region were imaged using swept-source OCT (DRI-OCT Triton, Topcon) with a central wavelength of 1050 nm and an acquisition speed of 100,000 A-scans per second (see Figure 1).

**Figure 2:**
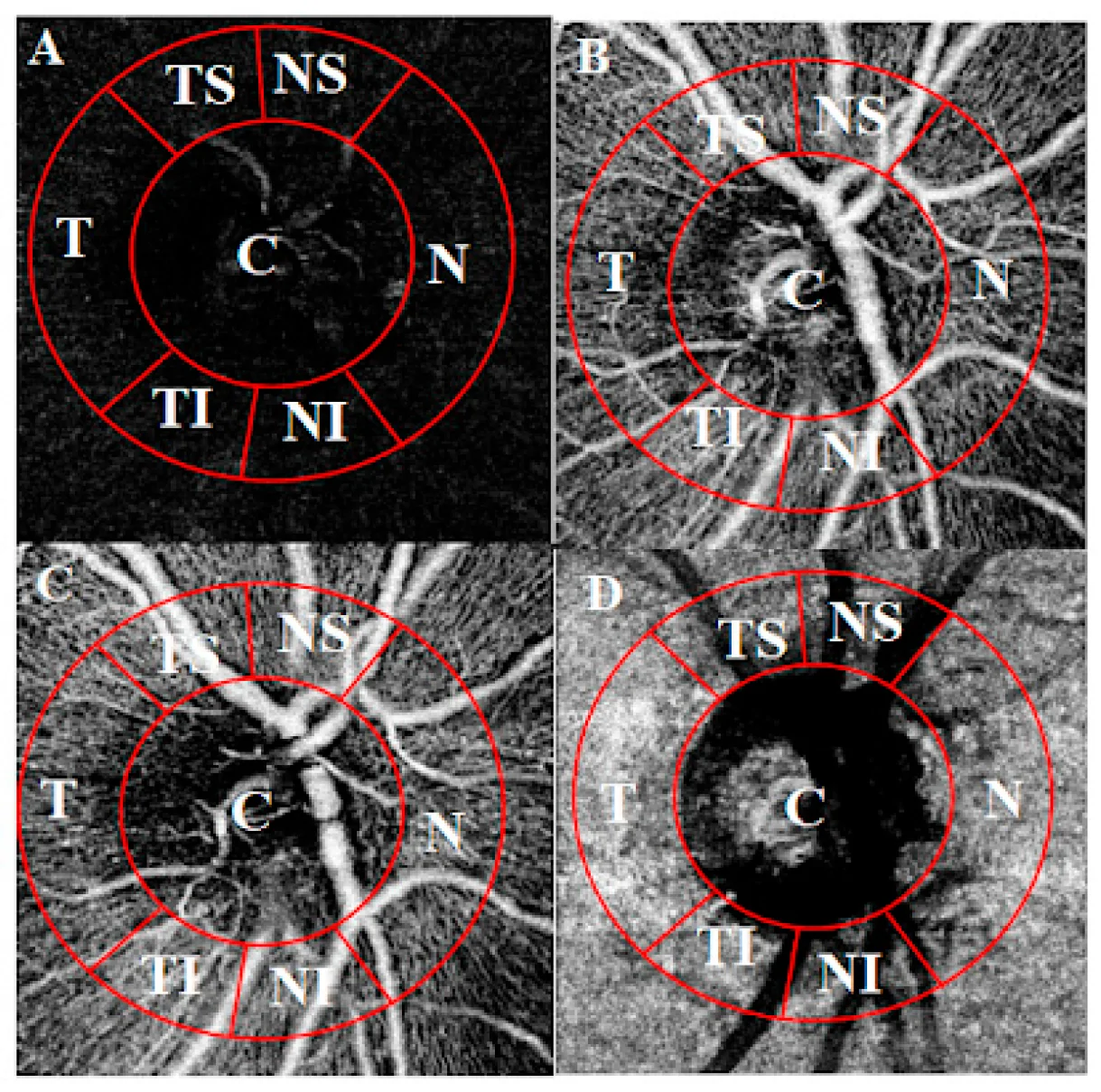
Optical coherence tomography angiography of optic nerve head (3 × 3 mm). Layers: (**A**)—superficial plexus, (**B**)—deep plexus, (**C**)—avascular retina, (**D**)—choriocapillaris. Sectors: C—central, NS—superior nasal, N—nasal, NI—inferior nasal, TI—inferior temporal, T—temporal, TS—superior temporal [10].

Vascular slabs were defined according to published OCTA segmentation recommendations [9]: the superficial capillary plexus from the internal limiting membrane + 2.6 µm to the IPL/INL boundary + 15.6 µm; the deep capillary plexus from the IPL/INL boundary + 15.6 µm to the IPL/INL boundary + 70.2 µm; the outer retina from the IPL/INL boundary + 70.2 µm to Bruch’s membrane; and the choriocapillaris from Bruch’s membrane to Bruch’s membrane + 10.4 µm. The outer retina slab, referred to here as the avascular retina, is physiologically devoid of vessels. Signal quantified within this slab therefore does not represent true VD and predominantly reflects projection artefact from the overlying plexuses together with background noise. Values from this slab are reported as a relative measure of projected signal rather than as perfusion. Projection artefact removal was not applied. OCTA VD was measured within a 750-µm wide circular annulus extending outward from the ONH border, divided into seven sectors (C, T, TS, NS, N, NI, TI), using ImageJ software (v1.53k; NIH, Bethesda, MA, USA) with Phansalkar thresholding and particle analysis, as previously described (see Figure 2) [10]. Each sector was measured in triplicate and averaged. Images with a quality score >40 were included. Eyes with insufficient image quality (e.g., blurring preventing vessel delineation) were excluded. One eye per participant was analyzed, selected at random and independently of disease severity and image quality. Because only one eye per participant was included, no adjustment for inter-eye correlation was required. Vascular layers were segmented automatically by the device software, and all segmentation boundaries were visually inspected by a single examiner. Scans with segmentation errors affecting the slab of interest were excluded from analyses of that layer, as occurred in one POAG patient in the superficial plexus. The four RNFL quadrants were matched to the OCTA sectors sharing the same anatomical location: the superior quadrant with the superior-nasal and superior-temporal sectors, the inferior quadrant with the inferior-nasal and inferior-temporal sectors, and the nasal and temporal quadrants with the corresponding single sectors. The central area was not matched to any RNFL quadrant.

fMRI was performed using a 3T Siemens Magnetom Skyra scanner (Siemens Healthineers, Erlangen, Germany) with a 32-channel head coil and a gradient-echo echo-planar imaging sequence with a repetition time of 3000 ms. A full-field reversing checkerboard was chosen because it produces a robust, well-characterized primary visual cortex response and does not depend on accurate fixation, which may be impaired in glaucoma. Participants viewed an alternating black-and-white checkerboard (2 Hz) binocularly during five 30-s stimulation blocks alternating with five 30-s rest blocks (100 dynamic scans per run, 5 min total). Two identical runs were acquired for each participant, and the resulting activity estimates were averaged. Preprocessing (motion correction, slice-timing correction, spatial smoothing with a 6 mm FWHM isotropic Gaussian kernel, normalization to MNI-152 space) and statistical analysis (general linear model with canonical hemodynamic response function) were performed using SPM8 (Wellcome Trust Centre for Neuroimaging, University College London, UK). Individual maps were thresholded at *p* < 0.05 (family-wise error corrected, minimum cluster size 10 voxels); group comparison maps were thresholded at *p* < 0.001 (uncorrected, minimum cluster size 10 voxels). Suprathreshold voxels within the occipital region were converted to a point-cloud representation in MATLAB (R2025b Update 1, The MathWorks Inc., Natick, MA, USA). The region of interest was defined by spherical approximation of the posterior skull, and spatial clustering was applied to isolate the main activation areas and exclude small foci remote from the expected location of the visual cortex. The sum of voxel intensities across clusters within this region was used as the measure of visual cortex activity, expressed in arbitrary units. Values from the two runs were averaged.

Statistical analyses were performed using Microsoft Excel 2010 and IBM SPSS Statistics 25 (IBM Corp., Armonk, NY, USA). Normality was assessed with the Shapiro–Wilk test. Groups were compared using Welch’s *t*-test, the Mann–Whitney U test where distributions were skewed, and Fisher’s exact test for categorical variables; the primary comparison is reported with its 95% confidence interval and Hedges’ g. Associations between RNFL thickness and VD were assessed with Pearson’s correlation, as these variables were normally distributed (Shapiro–Wilk *p* ≥ 0.09 for all pairs reported in Table 2). Correlations involving the visual cortex activity measure were assessed with Spearman’s rank correlation, as that variable was not normally distributed (Shapiro–Wilk *p* = 0.049). One hypothesis was prespecified—that RNFL thickness would correlate with VD in anatomically corresponding sectors—and all other analyses are exploratory, with *p* values adjusted by the Benjamini–Hochberg false discovery rate within each family of tests (24 RNFL-VD correlations, 28 VD-cortical activity correlations, and 28 between-group comparisons, each within group). The primary outcome and the prespecified correlations were also analyzed with adjustment for age. A two-sided *p* < 0.05 was considered significant.

## 3. Results

The baseline demographic and clinical results of the glaucoma and control groups are presented in Table 1.

POAG and control subjects were comparable with respect to sex distribution, BMI, systemic blood pressure, IOP, with no statistically significant differences observed. Participants in the glaucoma group were slightly older on average. POAG patients showed lower VFI, more negative MD and higher PSD, consistent with greater visual field damage. RNFL thickness was significantly reduced in the POAG group compared with controls across all measured sectors (all *p* < 0.05).

**Figure 3:**
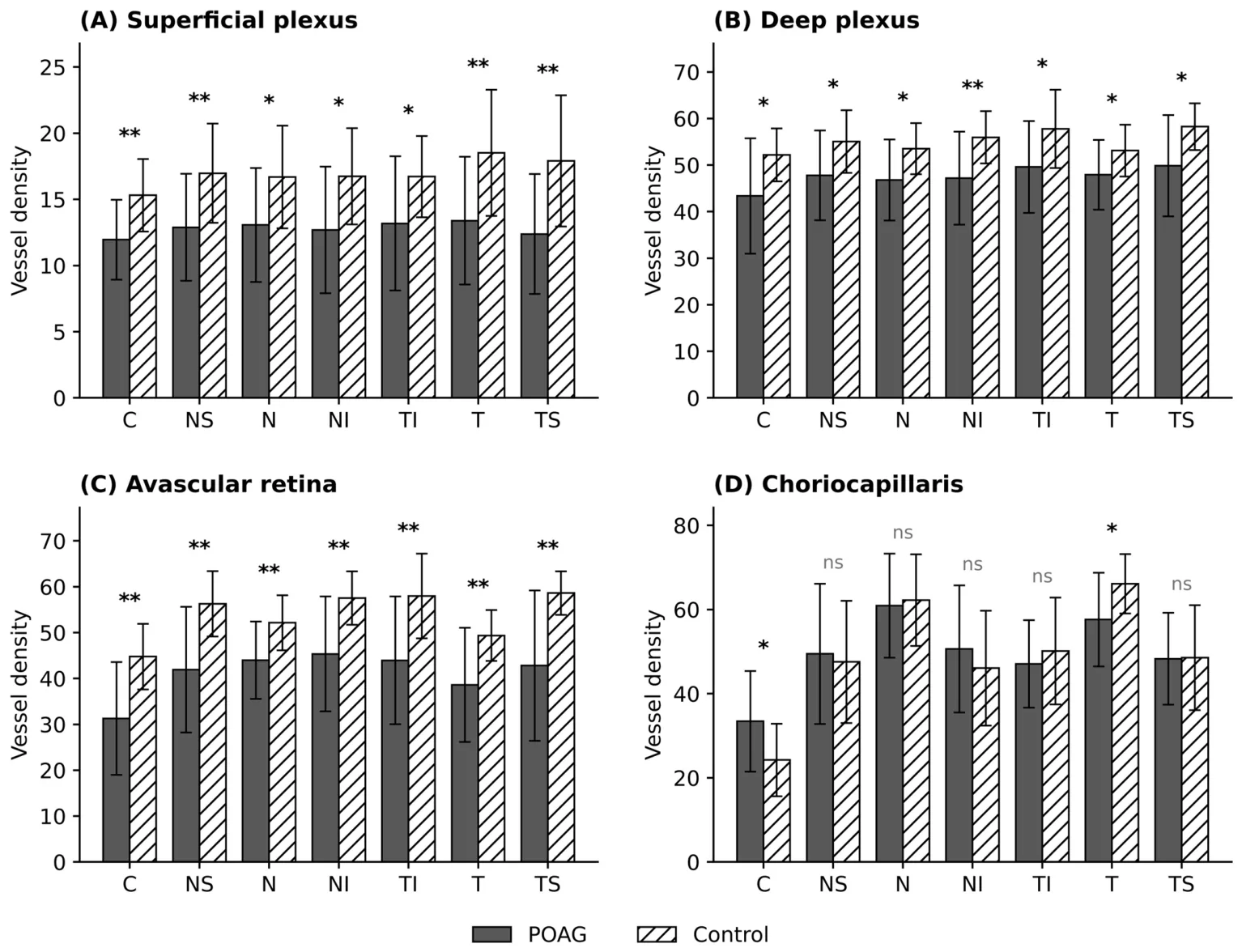
Differences of VD on OCTA scan of the ONH in the superficial plexus (**A**), deep plexus (**B**), outer retinal slab (**C**), labelled avascular retina; values represent projected signal, not perfusion) and choriocapillaris layer (**D**) in glaucoma and control groups. Significance markers denote Benjamini-Hochberg adjusted q values across all 28 between group comparisons: * q < 0.05, ** q < 0.01, ns—not significant. Sectors: C—central, NS—superior nasal, N—nasal, NI—inferior nasal, TI—inferior temporal, T—temporal, TS—superior temporal.

VD differences between groups across all four vascular layers are shown in Figure 3.

VD was lower in the POAG group in every sector of the superficial and deep plexus (all q < 0.05). The signal quantified in the outer-retinal (avascular) slab was likewise lower in every sector. The choriocapillaris showed a different pattern: VD was higher in the POAG group in the central area, lower in the temporal sector, and did not differ in the remaining sectors. One POAG patient was excluded from superficial plexus analyses because of a segmentation failure in the superficial slab (*n* = 18 for this layer).

To further characterize the relationship between structural and microvascular changes, correlations between RNFL thickness and VD were assessed within anatomically corresponding sectors across all four vascular layers (Table 2).

**Table 2:** Correlations between peripapillary RNFL thickness and choriocapillaris VD in anatomically corresponding sectors.

RNFL Sector	Corresponding VD Sector	Glaucoma r (*p*)	Glaucoma q	Control r (*p*)
Superior	Superior-temporal	0.256 (0.290)	0.968	−0.167 (0.509)
	Superior-nasal	**0.629 (0.004** **)**	**0.047**	0.190 (0.450)
Nasal	Nasal	**0.673 (0.002)**	**0.038**	−0.150 (0.553)
Inferior	Inferior-nasal	**0.586 (0.008)**	0.068	0.117 (0.643)
	Inferior-temporal	0.058 (0.815)	0.968	−0.180 (0.475)
Temporal	Temporal	−0.265 (0.273)	0.968	−0.159 (0.528)

The significant correlations stay in bold.

Positive correlations between RNFL thickness and choriocapillaris VD were observed in the nasal, superior-nasal and inferior-nasal sectors in POAG patients. After FDR correction, the nasal and superior-nasal associations remained significant, whereas the inferior-nasal association did not (q = 0.068). After adjustment for age, the partial correlations were 0.693 (95% CI 0.33 to 0.88, *p* = 0.001) for the superior-nasal sector, 0.673 (95% CI 0.30 to 0.87, *p* = 0.002) for the nasal sector and 0.576 (95% CI 0.15 to 0.82, *p* = 0.010) for the inferior-nasal sector. After FDR correction within the same family of 24 tests, the superior-nasal and nasal associations remained significant (q = 0.019 for both), whereas the inferior-nasal association did not (q = 0.078). In the remaining three sectors, the partial correlations were 0.306 (*p* = 0.203), 0.121 (*p* = 0.622) and −0.272 (*p* = 0.260). In the control group, no correlation was significant before or after correction (all q ≥ 0.603). Complete results for all four layers are given in Supplementary Table S1.

**Figure 4:**
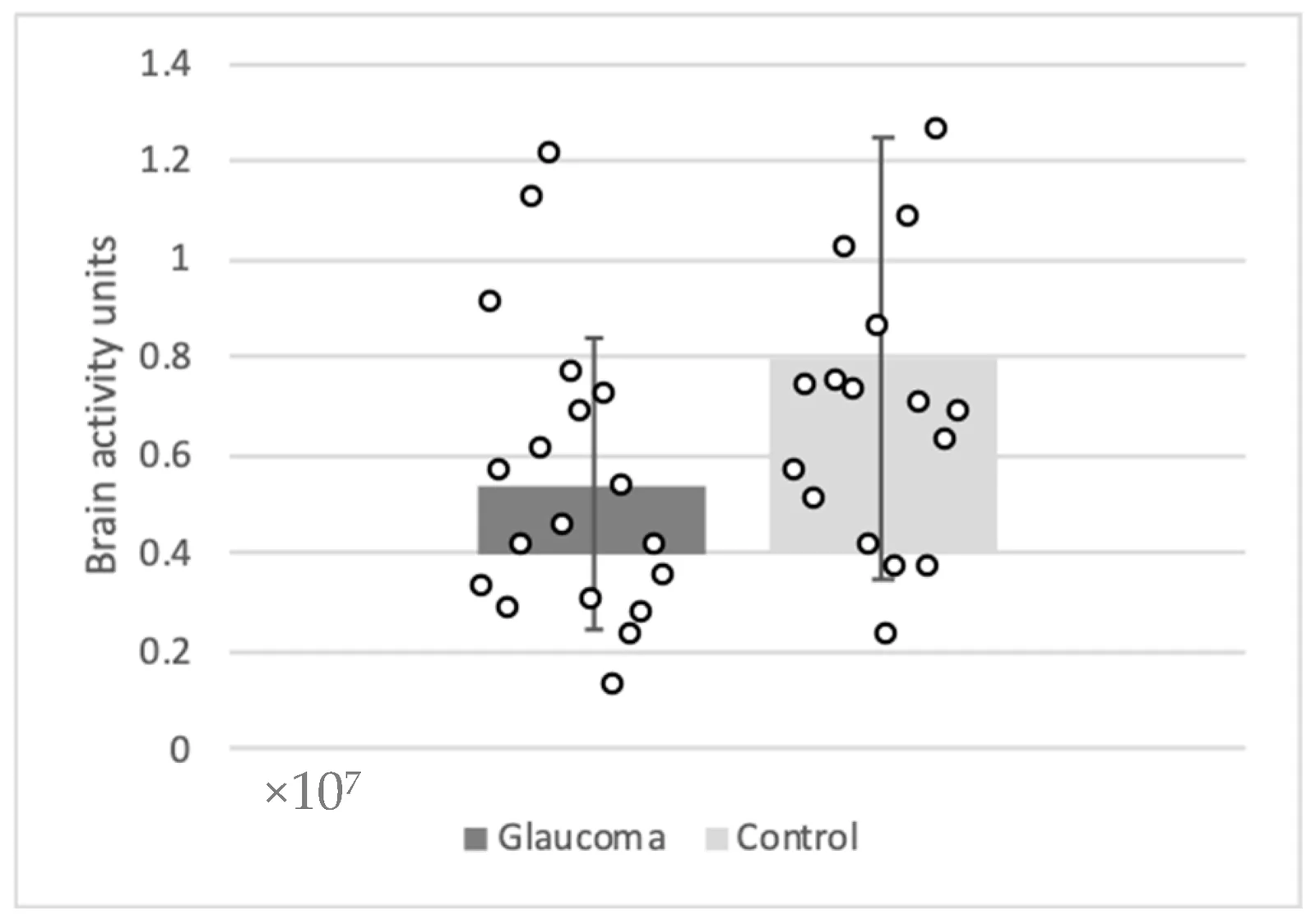
Comparison of brain activity units in glaucoma and control groups.

Visual cortex activity was compared between groups, as shown in Figure 4.

Visual cortex activation was 5.39 × 10^6^ arbitrary units in the POAG group and 8.01 × 10^6^ in controls (*p* = 0.046; Hedges’ g = −0.68, 95% CI −1.34 to −0.01). After adjustment for age, the between-group difference was −2.36 × 10^6^ arbitrary units (95% CI −5.08 × 10^6^ to +0.36 × 10^6^, *p* = 0.086).

Associations between visual cortex activity and VD were examined in all four vascular layers across seven sectors (28 tests per group). In the POAG group, no correlation with superficial plexus VD reached significance (rho = 0.00 to −0.45, all *p* ≥ 0.062). Two correlations reached nominal significance, both in the deep plexus (central area rho = 0.49, *p* = 0.032; superior-nasal sector rho = 0.52, *p* = 0.024), but neither survived FDR correction (both q = 0.26), a rate consistent with chance given the number of tests performed. In the control group, no correlation reached nominal significance in any layer (rho = −0.33 to 0.42). Complete results are given in Supplementary Table S2.

## 4. Discussion

The main finding of this study was that choriocapillaris VD correlated with RNFL thickness in anatomically corresponding nasal sectors in patients with POAG, an association not detected in healthy controls and not present in the other three vascular layers. Visual cortex activation was lower in the POAG group, although this difference did not persist after adjustment for age, and we found no association between retinal VD and cortical activity in either group.

Reduced visual cortex activation in our POAG cohort (*p* = 0.046, attenuated after adjustment for age) aligns with a growing body of functional neuroimaging literature [2,11]. Zhou et al. reported reduced blood oxygen level-dependent (BOLD) responses in peripheral visual field representations together with evidence of functional remapping within the visual cortex of patients with mild-to-moderate glaucoma, suggesting that cortical functional reorganization may occur during disease progression. Furthermore, significant associations between cortical activity, RNFL thickness, and visual field indices were observed, supporting the relationship between retinal structural damage and cortical dysfunction [12]. Qing et al. demonstrated significantly reduced BOLD responses within the primary visual cortex in POAG patients despite preservation of central visual fields, suggesting that cortical functional abnormalities may already be present before severe visual impairment becomes clinically evident [13]. Similarly, Wang et al. demonstrated altered cerebral perfusion and disrupted functional connectivity involving visual cortical regions even in relatively early glaucoma stages, indicating that cerebral functional abnormalities may develop before advanced visual loss becomes clinically apparent [14].

Recent neuroimaging studies suggest that glaucomatous cortical alterations may involve adaptive functional reorganization in addition to neurodegeneration. Li et al. demonstrated disrupted effective connectivity between primary and higher-order visual cortices together with evidence of enhanced top-down processing and cortical network reorganization in patients with normal-tension glaucoma (NTG). The authors proposed that increased connectivity from higher visual cortices to primary visual regions may represent a compensatory response to progressive lower visual pathway injury [15].

Similarly, the reduced RNFL thickness and decreased VD across multiple retinal layers observed in our POAG group are consistent with prior OCT and OCTA studies [6,8], reinforcing established structural and microvascular hallmarks of glaucomatous damage.

A more specific finding was that RNFL thickness correlated significantly with VD within the choriocapillaris layer—specifically in the nasal, superior-nasal, and inferior-nasal sectors—in POAG patients but not controls. After correction for multiple comparisons the nasal and superior-nasal associations remained significant, whereas the inferior-nasal association did not. No sector-corresponding correlations were observed in the superficial plexus, deep plexus or avascular retina in either group. Findings in the avascular retina slab should be interpreted with particular caution, as this slab contains no retinal vessels and the quantified signal reflects a projected signal from the overlying plexuses rather than perfusion. This selective association is consistent with prior work showing that choroidal microvasculature impairment tracks the rate of RNFL thinning in glaucoma, an effect attributed to the distinct blood supply of the superficial retina (central retinal artery) versus the choroid and choriocapillaris (short posterior ciliary artery) [16]. Our findings extend this concept by showing that the association is not only present in disease progression but also detectable cross-sectionally and is spatially specific to the nasal sectors in our cohort. The concentration of these associations in the nasal sectors may relate to the topography of the peripapillary choroidal supply from the short posterior ciliary arteries, which is not uniform around the disc, and to the fact that the nasal peripapillary region is less affected by large retinal vessel trunks that can interfere with thresholding-based VD measurement. With the present sample size, however, we cannot exclude that this spatial pattern arose by chance, and to our knowledge it has not been consistently reported previously.

We did not detect an association between retinal VD and visual cortex activation. Several explanations are possible. First, no such relationship may exist at this stage of disease, or it may not be captured by the measures used here. Second, statistical power was limited: at this sample size a correlation must exceed approximately 0.46 to reach significance. Third, fixation stability during the fMRI paradigm was not monitored, and differences in fixation between patients with more and less advanced disease cannot be excluded as a source of variability. Minosse et al. reported associations between brain connectivity measures, RNFL thickness and visual field indices [17], suggesting that such relationships may be detectable with network-level rather than activation-amplitude measures.

Taken together, these findings are compatible with choriocapillaris perfusion tracking local structural damage at the optic nerve head, while the reduction in cortical activation appears to occur independently of the retinal microvascular measures assessed here.

### Limitations

This study has several limitations. First, the sample was small and was powered to detect a standardized effect size of 0.95. The observed effect for cortical activation was smaller and the correlation analyses were underpowered. Second, the groups differed in age, and the between-group difference in cortical activation did not remain significant after adjustment. Third, apart from one prespecified hypothesis, all analyses were exploratory and are reported with FDR correction. Individual findings require replication, and non-significant findings should be interpreted as not detected rather than absent. Disease duration varied widely (range 0.5 to 16 years) and topical treatment was not standardized. Both could influence retinal microvasculature and cortical activity, and the sample was too small to examine them as covariates. In addition, VD in the avascular retina slab does not represent true perfusion, and projection artefact removal was not applied. The fMRI outcome was derived from the summed intensity of suprathreshold voxels, a measure that depends on the chosen statistical threshold and conflates the spatial extent and amplitude of activation. This was a cross-sectional, single center study, which cannot establish the temporal sequence of retinal and cortical changes. No causal or adaptive interpretation can be drawn from the associations reported here.

## 5. Conclusions

In POAG, choriocapillaris VD correlated with RNFL thickness in anatomically corresponding nasal sectors, an association not detected in healthy controls. Visual cortex activation was lower in POAG, although this difference was attenuated after adjustment for age, and no association between retinal VD and cortical activity was found. These exploratory findings require confirmation in larger, adequately powered cohorts before any relationship between retinal microvascular and cortical measures can be inferred. Further longitudinal studies are needed to clarify the temporal relationship between retinal microvascular change and cortical functional change across the full spectrum of glaucoma severity.

## Figures and Tables

**Table 1:** Demographic and ocular characteristics in glaucoma patients and healthy controls.

Parameter	Glaucoma Group (*n* = 19) Mean (SD)	Control Group (*n* = 18) Mean (SD)	*p*-Value
Age (years)	62.26 (4.6)	58.56 (5.68)	0.037
Female:Male ratio	11:8	13:5	0.495
Body mass index (BMI) (kg/m^2^)	28.47 (5.25)	27.95 (5.17)	0.764
IOP (mmHg)	14.88 (4.18)	14.51 (2.2)	0.739
Systolic blood pressure (mmHg)	134.95 (17.93)	138.78 (21.2)	0.558
Diastolic blood pressure (mmHg)	83.68 (15.52)	77.67 (8.11)	0.148
MD	−6.73 (6.79)	−0.94 (1.41)	0.002
PSD	6.84 (4.8)	1.87 (0.83)	<0.001
VFI	82.63 (18.81)	98.61 (1.38)	0.002
RNFL superior (µm)	91.61 (32.04)	126.93 (12.08)	<0.001
RNFL nasal (µm)	69.91 (16.04)	80.80 (14.18)	0.035
RNFL inferior (µm)	91.63 (31.82)	132.50 (13.47)	<0.001
RNFL temporal (µm)	59.40 (18.84)	74.46 (9.77)	0.005

Between-group comparisons used Welch’s *t*-test, except sex distribution (Fisher’s exact test). MD, PSD and VFI refer to the studied eye.

## Data Availability

Dataset available on request from the authors.

## Supplementary Materials

The following supporting information can be downloaded at: https://www.mdpi.com/article/10.3390/medicina62102018/s1, Table S1. Pearson correlations between peripapillary RNFL thickness and vessel density in anatomically corresponding sectors, all four vascular layers. Table S2. Spearman correlations between visual cortex activity and vessel density, all four vascular layers and seven sectors.

## Abbreviations

The following abbreviations are used in this manuscript:

BCVA	best-corrected visual acuity
BMI	body mass index
BOLD	blood oxygen level-dependent
CCT	central corneal thickness
CNS	central nervous system
fMRI	functional magnetic resonance imaging
IOP	intraocular pressure
MD	mean deviation
POAG	primary open-angle glaucoma
OCT	optical coherence tomography
OCTA	optical coherence tomography angiography
ONH	optic nerve head
PSD	pattern standard deviation
RGC	retinal ganglion cell
RNFL	retinal nerve fiber layer
VD	vessel density
VFI	visual field index
