# Supplementary material for: Visual Cortex Activity and Retinal Changes in Primary Open-Angle Glaucoma: An fMRI and OCTA Study
Source: Medicina (Kaunas). 2026 Oct 19;62(10):2018. doi: 10.3390/medicina62102018 (PMC13706879; doi:10.3390/medicina62102018)
Supplement: Supplementary file 1 [file medicina-62-02018-s001.zip › Table S1.pdf]

Table S1. Pearson correlations between peripapillary RNFL thickness and vessel density in anatomically corresponding sectors, all four vascular layers.						
**Group**	**Layer**	**RNFL / VD sector**	**n**	**r**	**p**	**q**
Glaucoma	Superficial plexus	Superior / TS	18	-0.204	0.417	0.937
		Superior / NS	18	-0.235	0.347	0.937
		Nasal / N	18	0.148	0.558	0.948
		Inferior / NI	18	0.078	0.758	0.948
		Inferior / TI	18	-0.04	0.875	0.954
		Temporal / T	18	-0.467	0.05	0.303
	Deep plexus	Superior / TS	19	0.244	0.315	0.937
		Superior / NS	19	0.092	0.707	0.948
		Nasal / N	19	0.207	0.394	0.937
		Inferior / NI	19	0.07	0.775	0.948
		Inferior / TI	19	0.193	0.43	0.937
		Temporal / T	19	-0.053	0.83	0.948
	Avascular retina	Superior / TS	19	-0.022	0.93	0.965
		Superior / NS	19	-0.122	0.618	0.948
		Nasal / N	19	0.067	0.786	0.948
		Inferior / NI	19	0.131	0.592	0.948
		Inferior / TI	19	0.011	0.965	0.965
		Temporal / T	19	-0.143	0.558	0.948
	Choriocapillaris	Superior / TS	19	0.256	0.29	0.937
		Superior / NS	19	0.629	0.004	0.047
		Nasal / N	19	0.673	0.002	0.038
		Inferior / NI	19	0.586	0.008	0.068
		Inferior / TI	19	0.058	0.815	0.948
		Temporal / T	19	-0.265	0.273	0.937
Control	Superficial plexus	Superior / TS	18	0.265	0.288	0.603
		Superior / NS	18	0.199	0.428	0.603
		Nasal / N	18	-0.18	0.474	0.603
		Inferior / NI	18	-0.388	0.112	0.603
		Inferior / TI	18	-0.446	0.064	0.603
		Temporal / T	18	0.177	0.482	0.603
	Deep plexus	Superior / TS	18	-0.209	0.405	0.603
		Superior / NS	18	-0.362	0.14	0.603
		Nasal / N	18	-0.282	0.257	0.603
		Inferior / NI	18	-0.232	0.354	0.603
		Inferior / TI	18	-0.248	0.322	0.603
		Temporal / T	18	0.177	0.483	0.603
	Avascular retina	Superior / TS	18	-0.306	0.216	0.603
		Superior / NS	18	-0.384	0.116	0.603
		Nasal / N	18	-0.481	0.043	0.603
		Inferior / NI	18	-0.176	0.485	0.603
		Inferior / TI	18	-0.23	0.359	0.603

		Temporal / T	18	0.045	0.859	0.859
	Choriocapillaris	Superior / TS	18	-0.167	0.509	0.603
		Superior / NS	18	0.19	0.45	0.603
		Nasal / N	18	-0.15	0.553	0.603
		Inferior / NI	18	0.117	0.643	0.671
		Inferior / TI	18	-0.18	0.475	0.603
		Temporal / T	18	-0.159	0.528	0.603
