# Supplementary material for: Visual Cortex Activity and Retinal Changes in Primary Open-Angle Glaucoma: An fMRI and OCTA Study
Source: Medicina (Kaunas). 2026 Oct 19;62(10):2018. doi: 10.3390/medicina62102018 (PMC13706879; doi:10.3390/medicina62102018)
Supplement: Supplementary file 1 [file medicina-62-02018-s001.zip › Table S2.pdf]

Table S2. Spearman correlations between visual cortex activity and vessel density, all four vascular layers and seven sectors.						
**Group**	**Layer**	**Sector**	**n**	**rho**	**p**	**q**
Glaucoma	Superficial plexus	C	18	-0.449	0.062	0.259
		NS	18	0.003	0.99	0.99
		N	18	-0.418	0.084	0.262
		NI	18	-0.082	0.748	0.798
		TI	18	-0.265	0.287	0.537
		T	18	-0.344	0.163	0.427
		TS	18	-0.082	0.748	0.798
	Deep plexus	C	19	0.493	0.032	0.259
		NS	19	0.516	0.024	0.259
		N	19	0.293	0.223	0.447
		NI	19	0.428	0.067	0.259
		TI	19	0.419	0.074	0.259
		T	19	0.16	0.514	0.685
		TS	19	0.179	0.464	0.649
	Avascular retina	C	19	0.425	0.07	0.259
		NS	19	0.419	0.074	0.259
		N	19	0.43	0.066	0.259
		NI	19	0.293	0.223	0.447
		TI	19	0.125	0.611	0.744
		T	19	0.204	0.403	0.607
		TS	19	0.109	0.658	0.767
	Choriocapillaris	C	19	0.218	0.371	0.607
		NS	19	0.14	0.567	0.721
		N	19	0.33	0.168	0.427
		NI	19	0.204	0.403	0.607
		TI	19	-0.305	0.204	0.447
		T	19	-0.2	0.412	0.607
		TS	19	-0.072	0.77	0.798
Control	Superficial plexus	C	18	-0.302	0.223	0.806
		NS	18	-0.309	0.213	0.806
		N	18	-0.263	0.291	0.806
		NI	18	-0.067	0.791	0.923
		TI	18	-0.257	0.303	0.806
		T	18	-0.135	0.593	0.806
		TS	18	-0.325	0.188	0.806
	Deep plexus	C	18	0.067	0.791	0.923
		NS	18	0.162	0.521	0.806
		N	18	-0.015	0.951	0.99
		NI	18	0.191	0.448	0.806
		TI	18	0.319	0.197	0.806
		T	18	0.23	0.358	0.806

		TS	18	0.143	0.57	0.806
	Avascular retina	C	18	0.003	0.99	0.99
		NS	18	0.131	0.604	0.806
		N	18	0.181	0.473	0.806
		NI	18	0.269	0.28	0.806
		TI	18	0.418	0.084	0.806
		T	18	0.226	0.367	0.806
		TS	18	0.176	0.484	0.806
	Choriocapillaris	C	18	0.29	0.243	0.806
		NS	18	-0.189	0.453	0.806
		N	18	0.022	0.932	0.99
		NI	18	0.395	0.104	0.806
		TI	18	-0.009	0.971	0.99
		T	18	-0.071	0.779	0.923
		TS	18	-0.152	0.548	0.806
